# Pediatric continuous renal replacement therapy: have practice changes changed outcomes? A large single-center ten-year retrospective evaluation

**DOI:** 10.1186/s12882-018-1068-1

**Published:** 2018-10-19

**Authors:** Alyssa A. Riley, Mary Watson, Carolyn Smith, Danielle Guffey, Charles G. Minard, Helen Currier, Ayse Akcan Arikan

**Affiliations:** 1 Department of Pediatrics, Renal Section, Baylor College of Medicine, Houston, TX USA; 2 Texas Children’s Hospital, Houston, TX USA; 3 Department of Pediatrics, Dell Medical School, The University of Texas at Austin, Austin, TX USA; 4 Dan L. Duncan Institute for Clinical and Translational Research, Baylor College of Medicine, Houston, TX USA

**Keywords:** Acute kidney injury, Children, Pediatric, Continuous renal replacement therapy, Renal replacement therapy acute, Pediatric CRRT

## Abstract

**Background:**

To evaluate changes in population characteristics and outcomes in a large single-center pediatric patient cohort treated with continuous renal replacement therapy (CRRT) over a 10 year course, coincident with multiple institutional practice changes in CRRT delivery.

**Methods:**

A retrospective cohort study with comparative analysis of all patients treated from 2004 to 2013 with CRRT in the neonatal, pediatric, and cardiovascular intensive care units within a free-standing pediatric tertiary care hospital.

**Results:**

Three hundred eleven total patients were identified, 38 of whom received concurrent treatment with extracorporeal membrane oxygenation. 273 patients received CRRT only and were compared in two study eras (2004–2008 *n* = 129; 2009–2013 *n* = 144). Across eras, mean patient age decreased (9.2 vs 7.7 years, *p* = 0.08), and the most common principal diagnosis changed from cardiac to liver disease. There was an increase in patients treated with continuous renal replacement therapy between cohorts for acute kidney injury of multi factorial etiology (44% vs 56%) and a decrease in treated patients with sepsis (21% vs 11%, *p* = 0.04). There was no significant difference in survival to hospital discharge between eras (47% vs 49%). Improvement in outpatient follow-up after discharge amongst survivors was seen between study eras (33% vs 54%).

**Conclusions:**

Despite multiple institutional practice changes in provision of CRRT, few changes were seen regarding patient demographics, diseases treated, indications for therapy, and survival over 10 years at a single tertiary care. Recognition of need for follow-up nephrology care following CRRT is improving. Ongoing assessment of the patient population in a changing landscape of care for critically ill pediatric patients remains important.

## Background

Continuous renal replacement therapy (CRRT) has been established as the gold standard for management of critically ill pediatric patients with acute kidney injury (AKI) in resource replete settings. Provision of CRRT to pediatric patients was initially guided by extrapolation of adult data, as far more critically ill adult than pediatric patients require CRRT, allowing for larger cohort studies. Available pediatric data largely stem from the Prospective Pediatric CRRT (ppCRRT) Registry, a North American multi-center registry enrolling patients between 2001 through 2005. ppCRRT has been instrumental in describing demographics, outcomes, and practice patterns [1–11] for pediatric CRRT and has established CRRT as a safe and effective therapy in pediatric AKI. However, this registry predates the standardization of pediatric AKI definition, new generation of scale based machines and other improvements in the care of critically ill children such as lung protective ventilation strategies, sepsis care bundles, improvement in mechanical support device technology. In fact, pediatric critical illness mortality has decreased from 4.6% in the 1990s to a current rate of 2.4% [12, 13]. In addition, more recent awareness of an association between fluid overload and adverse outcomes, as well as the possibility that earlier CRRT starts portend a better prognosis may have resulted in a practice drift where CRRT is being started earlier at a lower cumulative fluid overload [14].

We hypothesized that with improvements and changes in delivery of critical care and parallel longitudinal improvements in delivery of CRRT, the demographics of CRRT patient population would change over time, not unlike changes previously reported in the epidemiology of pediatric AKI [15–19]. Additionally, we hypothesized that with the improved recognition of AKI and advances in critical care practice and CRRT technology, we would see a survival benefit over time. Our objectives were to determine if there were measurable changes in patient characteristics and outcomes over a 10-year course in a large single-center pediatric CRRT cohort, and to provide insight as to whether those changes, if any, could be related to institutional CRRT practice patterns.

## Methods

### Retrospective chart review

All patients who received CRRT, including neonatal, pediatric, and young adults, treated with CRRT at Texas Children’s Hospital from 2004 to 2013 were identified from an administrative database and included in the study. Study period was divided into two cohorts, from 2004 to 2008 and from 2009 to 2013, roughly paralleling several institutional practice changes to evaluate for difference in outcomes (Table 1). For patients having multiple CRRT courses, each unique hospitalization was considered as a separate patient case. The study was approved by the Baylor College of Medicine Institutional Review Board and informed consent was waived.

**Table 1:** Institutional changes affecting changes in the delivery process for pediatric continuous renal replacement therapy

• Equipment change from Prisma to Prismaflex	
• Standard prescription changed from CVVHD to CVVHDF	
• Standard anticoagulation changed from systemic heparin to regional citrate	
• Filter change from mostly AN69 to HF2000 membranes	
• Adoption of 24 h in-house dialysis nursing staff	
• Introduction of emergency department sepsis protocol^a^	
• Establishment of a dedicated Renal ICU physician team	
• Institutional CRRT Policies & Procedures manual written	
• Creation of an institutional CRRT prospective database	
• Creation of a prospective CRRT Quality Improvement Team	

CVVHD: continuous venovenous hemodialysis; CVVHDF: continuous venovenous hemodialfiltration

^a^ Akcan Arikan A, Williams EA, Graf JM, et al. Resuscitation Bundle in Pediatric Shock Decreases Acute Kidney Injury and Improves Outcomes. *J Pediatr*. Dec 2015;167(6):1301–1305

Medical records were reviewed to determine principal diagnosis, cause of AKI, indication for CRRT, and other clinical information. Additional data obtained based on hospital charges included sex, age at start of CRRT, total days and number of courses of CRRT, ventilation days, intensive care unit (ICU) and hospital length of stay (LOS), and discharge disposition. Patients were grouped by principal diagnosis categories, including renal disease with end stage renal disease, hematology/oncology diagnoses (excluding bone marrow transplantation), bone marrow transplant (BMT) recipients, cardiac disease (including heart transplant recipients), liver disease (including liver transplant recipients), pulmonary disease (including lung transplant recipients), primary sepsis without a pre-existing known underlying organ system disease, inborn errors of metabolism, neonatal, and other primary system involvement.

A primary etiology for kidney injury was determined based on review of nephrology and intensive care unit (ICU) physician documentation. Indications for CRRT were identified from nephrologists’ notes. Additional information recorded included patient’s ICU admission weight, concurrent provision of extracorporeal mechanical oxygenation (ECMO) or use of cardiac assist device, and failure of renal recovery at hospital discharge defined as ongoing need for dialysis. Percentage fluid overload prior to initiation of CRRT was determined as previously reported: [(fluid in) - (fluid out)/(ICU admission weight)] * 100 [20]. All CRRT treatments were performed using Prisma or the Prismaflex control unit (Gambro, Sweden and Baxter, USA),), with a standard initial prescription of 2000 ml/min/m2 divided equally between dialysate and replacement. Outpatient nephrology follow-up was defined as an outpatient visit within 12 months of hospital discharge with a Texas Children’s Hospital nephrologist in survivors.

### Statistical analysis

Summary statistics of patient characteristics are described using mean and standard deviation, median and 25th and 75th percentiles (IQR), or frequency and percentage. Summary statistics are stratified by era (2004–2008, 2009–2013) and compared using t-test, Wilcoxon rank-sum test, chi-square, or Fisher’s exact test. Variables related to time and fluid overload are stratified by era and discharge disposition and eras are compared among survivors and non-survivors separately. Survival was compared between and within era by principal diagnosis, weight, CCRT indication, and immune status using Fisher’s exact test. All analyses were performed using Stata version 12.1 (Stata Corp, College Station, TX).

## Results

A total of 311 patients were treated with CRRT during the study period, 38 of whom were concurrently treated on ECMO. Patient characteristics of the 10-year cohort are shown in Table 2, showing inclusion and exclusion of ECMO patients. From 2004 to 2008, 129 individual patients, and from 2009 to 2013, 144 individual patients were treated with CRRT (Table 3). The institutional volume of CRRT increased 12% (15 patients) between the two eras. Three patients in the 2004–2008 cohort and 4 patients in the 2009–2013 cohort each had two separate hospitalizations during which they received CRRT, however for statistical analysis, each patient was only considered once in their respective cohorts, with the first observation of CRRT retained in statistical analysis. Although not statistically significant, patients in the latter cohort were younger than in the early cohort (7.7 vs. 9.2 years, *p* = 0.08). There was also a shift toward lower weight patients, including 12 more patients in the 0–10 kg group treated with CRRT from 2009 to 2013 (*n* = 38) than in 2004–2008 (*n* = 26), as well as 10 additional patients in the 10–20 kg treated from 2009 to 2013 (*n* = 39) compared with 29 in the 2004–2008 group. There was a modest reduction in percent fluid overload at initiation of CRRT (17% vs. 14%, *p* = 0.19). Survival to ICU discharge, 28-day survival, 60-day survival, survival to hospital discharge was comparable between groups. Follow-up with a nephrologist after hospital discharge was significantly improved over time (*p* < 0.05), with 54% of 60 surviving patients treated between 2009 and 2013 being seen in the outpatient nephrology clinic within one year of discharge. Four of the surviving patients were followed with nephrology due to reaching end-stage renal disease after critical illnesses, which were autoimmune hepatitis with fulminant hepatic failure, biliary atresia requiring liver transplant, Streptococcal pneumoniae sepsis with underlying sickle cell disease, and severe combined immunodeficiency syndrome. Table 3 also displays the principal diagnosis, causes of AKI, and indications for patients treated with CRRT in the two cohort groups. The constellation of principal diagnoses was similar between the two cohorts (*p* = 0.06), however some small shifts were seen in diagnoses treated, including more renal and liver patients, and fewer pulmonary and sepsis patients. The identified causes of AKI were different (*p* = 0.04) with a greater percentage of patients in the latter era having multifactorial AKI, and fewer patients considered having AKI caused solely by septic shock. Fluid overload was the most common indication for initiation of CRRT in both eras.

**Table 2:** Patient characteristics over the 10 year study period with and without ECMO patients

Characteristic, n (%)	All patients including ECMO	All patients excluding ECMO
Patients (n)	311	273
Age (n)		
0 to 1 yr.	73 (23%)	56 (21%)
1 to 3 yr	37 (12%)	34 (12%)
3 to 5 yr	23 (7%)	23 (8%)
5 to 10 yr	41 (13%)	38 (14%)
10 to 15 yr	63 (20%)	57 (21%)
15 to 21 yr	64 (21%)	55 (20%)
> 21 yr	10 (3%)	10 (4%)
Mean (SD)	8.2 (7.0)	8.4 (7.0)
Weight (n)		
0 to 10 kg	82 (26%)	64 (23%)
10 to 20 kg	71 (23%)	68 (25%)
20 to 50 kg	80 (26%)	68 (25%)
> 50 kg	78 (25%)	73 (27%)
Mean (SD)	33 (28.1)	33.5 (27.2)
Pre-existing end-stage renal disease	13 (4%)	13 (5%)
Sepsis on admission	36 (13%)	34 (14%)
Immunocompromised		
Solid organ transplant	67 (22%)	58 (21%)
BMT	33 (11%)	31 (11%)
Other immunocompromised	54 (17%)	51 (19%)
Not immunocompromised	156 (50%)	132 (49%)
% Fluid overload at CRRT Start ^a^	15 (7, 26)	15 (8, 26)
Hospital Unit of CRRT Start		
Pediatric Intensive Care Unit	257 (83%)	241 (89%)
Neonatal Intensive Care Unit	22 (7%)	14 (5%)
Cardiovascular Intensive Care Unit	29 (9%)	15 (6%)
Bone Marrow Transplant Unit	1 (0.3%)	1 (0.4%)
ICU survival	154 (50%)	145 (53%)
28 day survival	176 (57%)	165 (60%)
60 day survival	156 (50%)	146 (53%)
Survival to discharge	139 (45%)	131 (48%)
Outpatient Renal Follow-up ^b^	62 (45%)	58 (44%)

^a^Median with (interquartile range)

^b^Patients who received CRRT for non-renal indications were excluded from this cohort (inborn errors of metabolism, ingestion)

**Table 3:** Comparing patient characteristics, principal diagnoses, causes of acute kidney injury, and indications for CRRT between study eras (excluding ECMO patients)

Characteristic, n (%)	2004–2008	2009–2013	*p*-value
Patients (*n*)	129	144	
Age (*n*)			
0 to 1 yr.	24 (19%)	32 (22%)	
1 to 3 yr	15 (12%)	19 (13%)	
3 to 5 yr	7 (5%)	16 (11%)	
5 to 10 yr	19 (15%)	19 (13%)	
10 to 15 yr	28 (22%)	29 (20%)	
15 to 21 yr	29 (22%)	26 (18%)	
> 21 yr	7 (5%)	3 (2%)	
Mean (*SD*)	9.2 (7.2)	7.7 (6.8)	0.08
Weight (*n*)			
0 to 10 kg	26 (20%)	38 (26%)	
10 to 20 kg	29 (22%)	39 (27%)	
20 to 50 kg	35 (27%)	33 (23%)	
> 50 kg	39 (30%)	34 (24%)	
Mean (*SD*)	36.0 (28.6)	31.3 (25.7)	0.15
Pre-existing end-stage renal disease	8 (6%)	5 (3%)	0.40
Sepsis on admission ^a^	21 (20%)	13 (9%)	0.02
Immunocompromised			0.74
Solid organ transplant	25 (20%)	33 (23%)	
BMT	17 (13%)	14 (10%)	
Not immunocompromised	61 (48%)	71 (49%)	
Other immunocompromised	25 (20%)	26 (18%)	
% Fluid overload at CRRT Start ^b^	17 (10, 26)	14 (6, 26)	0.19
Hospital Unit at CRRT Start			0.85
Pediatric Intensive Care Unit	115 (91%)	126 (88%)	
Neonatal Intensive Care Unit	6 (5%)	8 (6%)	
Cardiovascular Intensive Care Unit	6 (5%)	9 (6%)	
Bone Marrow Transplant Unit	0	1 (1%)	
ICU survival	68 (53%)	77 (53%)	0.90
28 day survival	79 (61%)	86 (60%)	0.81
60 day survival	68 (53%)	78 (54%)	0.90
Survival to discharge	60 (47%)	71 (49%)	0.72
Outpatient Renal Follow-up ^c^	20 (33%)	38 (54%)	0.02
Diagnosis, n (%)			0.06
Cardiac	13 (10%)	11 (8%)	
Renal	9 (7%)	19 (13%)	
Liver	19 (15%)	37 (26%)	
Hematology/Oncology	18 (14%)	17 (12%)	
Post-Bone Marrow Transplant	18 (14%)	15 (10%)	
Pulmonary	11 (9%)	4 (3%)	
Inborn error of metabolism	5 (4%)	10 (7%)	
Sepsis	10 (8%)	5 (4%)	
Neonates	10 (8%)	13 (9%)	
Other ^d^	16 (12%)	13 (9%)	
Cause of acute kidney injury ^a, e^	(*n* = 113)	(*n* = 124)	0.04
Multifactorial	50 (44%)	69 (56%)	
Septic shock	24 (21%)	14 (11%)	
Renal	10 (9%)	7 (6%)	
Poor cardiac function	5 (5%)	6 (5%)	
Hepatorenal syndrome	5 (4%)	8 (6%)	
Nephrotoxic drugs	4 (3%)	5 (4%)	
Abdominal compartment syndrome	1 (1%)	8 (6%)	
Other ^f^	16 (13%)	7 (6%)	
Indication for CRRT	(n = 129)	(n = 144)	0.41
Fluid overload	74 (57%)	71 (49%)	
Electrolyte management	7 (5%)	9 (6%)	
Fluid overload and electrolyte management	28 (22%)	42 (29%)	
Prevent fluid overload/provide nutrition	6 (5%)	3 (2%)	
Other	14 (11%)	19 (13%)	
*Hemodynamic instability*	3 (21%)	4 (21%)	
*Hyperammonemia*	6 (43%)	12 (63%)	
*Ingestion*	3 (21%)	3 (16%)	
*End-stage renal disease*	2 (14%)	0	

^a^*p* < 0.05 comparing 2004–2008 with 2009–2013

^b^Median with (interquartile range)

^c^Patients who received CRRT for non-renal indications were excluded from this cohort (inborn errors of metabolism, ingestion)

^d^Includes rheumatology, gastroenterology, multiple organ, neurology, ingestions, hemorrhage, rhabdomyolysis, and non-accidental trauma

^e^Patients with end-stage renal disease and non-acute kidney injury indications for CRRT (i.e. inborn error of metabolism) are excluded

^f^Includes vasculitis, microangiopathy, rhabdomyolysis, tumor lysis, obstruction, cardiac arrest, and unknown

Outcomes on CRRT were evaluated by measurements of days to start CRRT from hospital and ICU admission, total CRRT days, ventilation days, and ICU and hospital length of stay (LOS) (Table 4). Non-survivors had a longer time to CRRT start from hospital admission (survivors 4 (1, 10) vs non-survivors 16 (4, 35) days, *p* < 0.001) and from ICU admission (survivors 3 (1, 6) vs 7 (2, 16) days, p < 0.001) than survivors. Hospital LOS was longer in survivors (survivors 42 (28, 71) vs. 32 (19, 54) days, *p* = 0.003), as expected, ICU LOS was not different. Non-survivors had more ventilation days compared to survivors across the entire 10-year study period (survivors 13 (4, 28) vs non-survivors 18 (8, 33) days, *p* = 0.02), except for the 2009–2013 period when non-survivors had fewer ventilation days than the non-survivors in the 2004–2008 period (*p* = 0.01). The total days on CRRT were not different between survivors and non-survivors or between eras. Patient hospital survival was also examined accounting for primary diagnosis, ICU admission weight, indication for CRRT, and immune status (Table 5). Patients with a hematologic/oncologic primary diagnosis (excluding patients who had undergone BMT) in the 2004–2008 cohort had fewer deaths (*p* = 0.04). Survival was not different when compared by patient weight at CRRT start, indication for CRRT, time on CRRT, and immune status.

**Table 4:** Comparison of survival on CRRT by median time from hospital admission to CRRT start, time from intensive care unit (ICU) admission to CRRT start, CRRT duration, total ventilation days, ICU days, and hospital length of stay (LOS) (median (inter-quartile range)) within and between study eras, as well as over the 10-year study period

	2004–2008			2009–2013			*2004–2013*		
Time Variable (days)	All Patients	Survivors	Non-survivors	All Patients	Survivors	Non-survivors	*All Patients*	*Survivors*	*Non-survivors*
Time from Hospital Admit to CRRT Start	8 (2, 22)	4 (2, 9)	18 ^a^ (4, 36)	7 (3, 22)	4 (1, 11)	14 ^a^ (4, 32)	*8 (2, 22)*	*4 (1, 10)*	*16* ^*a*^ *(4, 35)*
Time from ICU Admit to CRRT Start	4 (1, 10)	3 (1, 6)	9 ^a^ (2, 19)	5 (2, 12)	2 (1, 5)	4.5 ^a^ (3, 14)	*3 (1, 10)*	*3 (1, 6)*	*7* ^*a*^ *(2, 16)*
Total CRRT time	8 (4, 16)	7 (4, 13)	8 (4, 19)	8 (4, 18)	8 (4, 17)	8 (4, 18)	*8 (4, 17)*	*7 (4, 15)*	*8 (4, 18)*
Ventilation time	21 (9, 35)	19 (6, 31)	21 (11, 37)	12 (5, 29)	11 (4, 25)	13^b^ (7, 30)	*16 (7, 31)*	*13 (4, 28)*	*18* ^*a*^ *(8, 33)*
ICU LOS	22 (13, 36)	21 (13, 34)	22 (13, 38)	19 (11, 33)	20 (11, 33)	19 (10, 33)	*21 (12, 34)*	*21 (12, 34)*	*21 (11, 36)*
Hospital LOS	39 (24, 63)	43 (31, 77)	36 ^a^ (19, 59)	35 (23, 60)	41 (26, 71)	31 ^a^ (20, 51)	*38 (23, 61)*	*42 (28, 71)*	*32* ^*a*^ *(19, 54)*

^a^*p* < 0.05 comparing survivors with non-survivors within the respective period cohort

^b^*p* < 0.05 comparing non-survivors from 2004 to 2008 with non-survivors from 2009 to 2013

**Table 5:** Comparison of survival on CRRT by principal diagnosis, weight, CRRT indication, and immune status between study eras and shown over the 10 year study period

	2004–2008	2009–2013	*2004–2013*
	Survivor n (%)	Survivor n (%)	*Survivor* *n (%)*
Principal Diagnosis Category			
Cardiac	7 (54%)	3 (27%)	*10 (42%)*
Renal	4 (44%)	14 (74%)	*18 (64%)*
Liver	6 (32%)	17 (46%)	*23 (41%)*
Hematology/Oncology ^a^	10 (56%)	3 (18%)	*13 (37%)*
Bone marrow transplant	3 (17%)	5 (33%)	*8 (24%)*
Pulmonary	4 (36%)	2 (50%)	*6 (40%)*
Inborn error of metabolism	5 (100%)	7 (70%)	*12 (80%)*
Sepsis	3 (30%)	2 (40%)	*5 (33%)*
Neonates	8 (80%)	8 (62%)	*16 (70%)*
Other ^b^	10 (63%)	10 (77%)	*20 (69%)*
Weight			
0-10 kg	9 (35%)	19 (50%)	*28 (44%)*
10-25 kg	13 (45%)	22 (56%)	*35 (51%)*
25-50 kg	17 (49%)	13 (39%)	*30 (44%)*
> 50 kg	21 (54%)	17 (50%)	*38 (52%)*
Indications for CRRT			
Fluid overload	31 (42%)	35 (49%)	*66 (46%)*
Fluid overload & electrolyte management	12 (43%)	17 (41%)	*29 (41%)*
Prevent fluid overload/provide nutrition	3 (50%)	2 (67%)	*5 (56%)*
Electrolyte management	4 (57%)	4 (44%)	*8 (50%)*
Other ^c^	10 (71%)	13 (68%)	*23 (70%)*
Days on CRRT			
1 day	8 (47%)	5 (55%)	*12 (50%)*
2–7 days	24 (42%)	34 (45%)	*58 (44%)*
8–14 days	18 (50%)	12 (36%)	*30 (43%)*
15–21 days	10 (53%)	11 (48%)	*21 (50%)*
22–28 days	1 (17%)	7 (50%)	*8 (40%)*
> 28 days	3 (25%)	7 (50%)	*10 (38%)*
Immune Status			
Solid organ transplant	11 (44%)	17 (52%)	*28 (48%)*
Bone marrow transplant	5 (29%)	5 (36%)	*10 (32%)*
Other immunocompromised	12 (48%)	11 (42%)	*23 (45%)*
Not immunocompromised	32 (52%)	38 (54%)	*70 (53%)*

^a^*p* < 0.05 comparing 2004–2008 survivors with 2009–2013 survivors

^b^Includes rheumatologic, gastroenterologic, multiple organ, neurologic, ingestions, hemorrhage, rhabdomyolysis, and non-accidental trauma

^c^Includes hemodynamic instability, hyperammonemia, ingestion, and end-stage renal disease

Table 6 shows a comparison of percent fluid overload at initiation of CRRT between eras based on principal diagnoses overall and survival. BMT patients were significantly less fluid overloaded upon initiation of CRRT (2004–2008 16% (10, 20) vs 2009–2013 10% (7, 12), *p* = 0.047), however there was no difference in survival. When the entire 10-year cohort was evaluated, sepsis survivors had lower percent fluid overload at initiation of CRRT (8% (12, 21)) compared with non-survivors (27% (19, 34)) (*p* = 0.05), however this was not demonstrated between the two eras. Neonatal survivors in the 2009–2013 cohort group also had lower percentage of fluid overload (17% (12, 22)) versus non-survivors (26% (14, 37)) (*p* = 0.043). No other differences were seen in the patient survival based on fluid overload and principal diagnosis.

**Table 6:** Comparison of median percent fluid overload upon initiation of CRRT in all patients, survivors, and non-survivors between eras (median (inter-quartile range)), as well as over the 10-year study period

Principal Diagnosis	2004–2008			2009–2013			*2004–2013*		
	All Patients	Survivors	Non-survivors	All Patients	Survivors	Non-survivors	*All Patients*	*Survivors*	*Non-survivors*
Cardiac	19 (2, 26)	5 (2, 22)	20 (19, 33)	4 (3, 11)	12 (3,29)	4 (0, 7)	*7 (2, 22)*	*9 (3, 24)*	*7 (0, 20)*
Renal	10 (6, 30)	10 (6, 30)	–	23 (13, 30)	17 (13, 30)	28 (28,28)	*20 (12, 30)*	*17 (11, 30)*	*28 (28, 28)*
Liver	20 (15, 31)	37 (17, 42)	18 (14, 24)	20 (12, 30)	19 (12, 30)	20 (12, 30)	*20 (12, 30)*	*20 (12, 35)*	*19 (13, 27)*
Hematology/ Oncology	15 (8, 22)	20 (12, 22)	12 (8, 15)	17 (5, 29)	14 (0, 29)	21 (5, 29)	*17 (8, 28)*	*17 (8, 22)*	*16 (8, 29)*
Bone marrow transplant	16 (10, 20)	15 (9,20)	16 (10, 20)	10 ^**a**^ (7, 12)	9 (7, 11)	10 (7, 13)	*11 (9, 18)*	*10 (8, 12)*	*11 (10, 19)*
Pulmonary	15 (9, 18)	20 (5, 26)	15 (11, 15)	13 (4, 22)	13 (8, 18)	13 (0, 25)	*15 (7, 19)*	*18 (8, 20)*	*15 (7, 16)*
Inborn Error of Metabolism	0 (0, 0)	0 (0, 0)	–	0 (0, 16)	0 (0, 0)	17 (0, 35)	*0 (0, 16)*	*0 (0, 0)*	*17 (0, 35)*
Sepsis	20 (9, 28)	8 (0, 21)	25 (13, 30)	22 (20, 38)	17 (12, 22)	38 (20, 46)	*21 (12, 30)*	*12 (8, 21)*	*27* ^***b***^ *(19, 34)*
Neonates	20 (9, 32)	21 (8, 39)	20 (20, 20)	14 (0, 26)	6 (0, 14)	26 ^**b**^ (14, 37)	*14 (6, 29)*	*10 (0, 15)*	*23 (14, 37)*
Other ^**c**^	21 (13, 40)	21 (13, 28)	28 (12, 48)	11 (4, 21)	17 (5, 21)	4 (0, 12)	*17 (8, 30)*	*17 (10, 28)*	*12 (4, 36)*

^a^ p < 0.05 comparing all patient median percent fluid overload between the 2004–2008 and 2009–2013 cohorts

^b^ *p* ≤ 0.05 comparing median percent fluid overload between survivors and non-survivors within the respective cohort period

^c^ Includes rheumatologic, gastroenterologic, multiple organ, neurologic, ingestions, hemorrhage, rhabdomyolysis, and non-accidental trauma

## Discussion

This cohort study of 311 patients treated with CRRT over a 10-year period is the largest single center pediatric report published to date. To our surprise, we found only a 12% increase in treatment volume over time and very similar patient characteristics in the two eras. There was a shift towards treatment of younger patients by 2.5 years, as well a nearly 5 kg shift towards smaller patients. Overall, the mean weight and age of this patient population were similar to those of the ppCRRT registry studies (33.5 kg vs 34.3 kg (ppCRRT) and 8.4 yr. vs 8.5 yr. (ppCRRT), with similar interval subcategorizations (Table 7) [3, 6]. It is worth noting that there may be a small measure of overlap in the ppCRRT registry data with our patients, as patients from our institution in years 2004 and 2005 were enrolled in that registry. We were unable to identify which patients were enrolled, and ppCRRT was a voluntary registry for which only those patients who were consented were included, thus it is extremely unlikely that every patient from those years was also in ppCRRT. In the modern era of CRRT, nearly half (46%) the patients treated were younger than 5 years old. Additionally, 53% of our patients were less than 20 kg and more than a quarter were less than 10 kg. This may be due to improving recognition of AKI in younger patients, and increasing comfort with providing CRRT for neonatal patients. The shift towards smaller, younger patients also falls in line with more recently published single-center cohort studies, as shown in Table 7 [21–24].

**Table 7:** Comparative outcomes of present study with previously published pediatric CRRT studies; FO, fluid overload, EM electrolyte management

	Our Study	ppCRRT Registry ^a, b^	Spain ^c, d^	Birmingham ^e^	Alberta (CRRT only) ^f^
N	273	344	174	76	49
Weight					
Mean/Median Wt (kg)	33.5	34.3		19.5(2.5–150)	19.5 (5.5–45)
Less than 10 kg (%)	23	24	43		
10 to 20 kg (%)	25	20			
20 to 50 kg (%)	25	29			
> 50 kg (%)	27	27			
Age					
Mean / Median Age (yr)	8.4	8.5	4.3 (±5.3)	5.8 (0–17.8)	5.4 (0.3–13.8)
0 to 1 yr. (%)	21	20	43.7		
1 to 3 yr. (%)	12	13			
3 to 5 yr. (%)	8	8			
5 to 10 yr. (%)	14	17			
10 to 15 yr. (%)	21	19			
15 to 21 yr. (%)	20	20			
> 21 yr. (%)	4	3			
Survival to discharge (%)	48	58	64	55	67
Principle diagnosis (%)					
Sepsis	5	24	20	12	
BMT	12	16		16	
Cardiac	9	12	56	9	
Renal	10	9	10	20	
Liver	21	8		6	
Malignancy	13	8		8	
Ischemia/shock		6			
Inborn error of metabolism	5	4	7		
Drug intoxication		4	2		
Tumor lysis syndrome		3	3	14	
Pulmonary	5	3			
Other	11	2	05	14	
Survival on CRRT by Weight (N (%))					
< 10 kg	28 (43%)	36 (43%)	57%		
> 10 kg	105 (49%)	165 (63%)	73%		
ICU days before CRRT	4 (1,10)	2			2 (1–5)
Days on CRRT	8 (4,16)				7 (3–18)
% FO	15 (8, 26)			12.9 (0–66.4)	20.1 (5.4–32.5)
Indication for CRRT & survival (%)					
FO and EM	29 (41%)	15	32^g^		
FO	67 (45%)	18			
EM	8(50%)	20	42^h^		
Prevent FO /provide nutrition	5(56%)	20			
Other	24 (55%)	21			

a. Symons JM, Chua AN, Somers MJ, et al. Demographic characteristics of pediatric continuous renal replacement therapy: a report of the prospective pediatric continuous renal replacement therapy registry. *Clin J Am Soc Nephrol*. July 2007;2(4):732–738

b. Sutherland SM, Zappitelli M, Alexander SR, et al. Fluid overload and mortality in children receiving continuous renal replacement therapy: the prospective pediatric continuous renal replacement therapy registry. *Am J Kidney Dis*. Feb 2010;55(2):316–25

c. Lopez-Herc J, Santiago MJ, Solana MJ et al. Clinical course of children requiring prolonged continuous renal replacement therapy. *Pediatr Nephrol*. Dec 2010; 25:523–528

d. Santiago MJ, Lopez-Herce J, Urbando J, et al. Clinical course and mortality risk factors in critically ill children requiring continuous renal replacement therapy. *Intensive Care Med*. May 2010;36(5):843–9

e. Hayes LW, Oster RA, Tofil NM, et al. Outcomes of critically ill children requiring continuous renal replacement therapy. *J Crit Care*. Sep 2009;24(3):394–400

f. Boschee E, Cave D, Garros D, et al. Indications and outcomes in children receiving renal replacement therapy in pediatric intensive care. *J Crit Care*. Feb 2014;29(1):37–42

g. Patients with diagnosis of AKI and hypervolemia

h. Patients with diagnosis of AKI

The shift towards younger, smaller patients continues to speak loudly to the need for equipment specially designed for treating the smallest patients, especially as our CRRT equipment is not United States Food and Drug Administration approved for use in patients weighing less than 20 kg. Following the ppCRRT evaluation, CRRT equipment evolved with the introduction of a new generation of machines now in use with better safeguards in place for delivery of CRRT to smaller patients [25]. A specialized pediatric CRRT circuit, the PrismaFlex HF20, with an extracorpeal volume of 60 ml [26] is currently being tested for FDA approval at pediatric centers throughout the USA. CARPEDIEM is RRT equipment with a 27 ml extracorpeal circuit volume and capability to handle blood flow rates 5–50 ml/hr., that once approved should dramatically improve the mechanics of treating the smallest patients [27, 28]. Additionally, the Newcastle infant dialysis and ultrafiltration system (NIDUS) is hemodialysis equipment designed for use in infants 800 g to 8 kg, without need for blood priming as well as using only single lumen vascular access, and successful use has been published on 9 babies weighing 1.8 kg to 5.9 kg in the United Kingdom [29]. There is additional work on pediatric equipment occurring, including the Aquadex™ continuous venovenous hemofiltration system [30], the KIDS-CRRT Device machine for pediatric fluid management [31], and a volumetric-based scale system for pediatric patients [32].

While not notably different, we did see shifts in the primary diagnoses of patients treated with CRRT in our institution over just 10 years. Across eras, there were double the number of patients treated with primary liver disease (19 vs 37) and nearly doubling of patients treated for inborn errors of metabolism (5 vs 10). This most likely reflects an institutional increase in tertiary referrals, where more patients with liver disease and inborn errors of metabolism are transported to our facility from around the region. Our institution does have an Extracorporeal Liver Support program; however, the cohort presented here predates the launch of that program so is unlikely to explain the increased numbers of liver failure patients placed on CRRT. Rather, this might be a reflection in the shift towards offering more CRRT to liver transplant candidates for AKI with or without hepatorenal syndrome, with the more recent reports of renal recovery after liver transplantation in pediatric and adult patients [33, 34]. Almost 3-fold fewer patients with pulmonary disease were treated with CRRT in the modern era (11 vs 4), which likely reflects the referral patients our center receives for its busy lung transplant program. A comparison of the primary diagnostic categories of published pediatric CRRT cohorts shown in Table 4 demonstrates the heterogeneity of the populations likely reflecting unique program strengths and referral regions. In our institution, cardiac population largely received acute peritoneal dialysis as the RRT of choice when possible. With the introduction of rasburicase, CRRT treatment for tumor lysis syndrome has become increasingly rare.

An important difference to note across the two eras was a significant decline in the number of patients treated with CRRT for an initial presentation of sepsis. We suspect this is largely due to the introduction of a septic shock protocol in early 2010, an early resuscitation bundle initiated upon patient presentation to the emergency department when meeting abnormal vital sign criteria within an electronic triage system to provide earlier recognition and treatment interventions [35]. The protocol has been shown to reduce AKI, the need for renal replacement therapy, hospital LOS, and patient mortality [36]. When evaluated alongside the ppCRRT registry and other single center studies, our center clearly has the smallest percentage of CRRT patients presenting with sepsis (Table 7). It is worth noting that the patient population with a primary diagnosis of sepsis was the only group to show a difference in percentage fluid overload between survivors and non-survivors when looking at the entire cohort over 10 years. An obvious predilection in either direction was not demonstrable for any other principal diagnoses. In many studies, the percentage of fluid overload at initiation of CRRT has repeatedly shown to be an independent risk factor for increased morbidity and mortality [3, 9, 20, 21, 37, 38], although conflicting reports also exist [39]. Awareness of the association between fluid overload and adverse outcomes has certainly increased, with pediatric data paving the way. In the initial report by Goldstein in 2001 [20], non-survivors of CRRT had almost 35% fluid overload at CRRT start. The lack of a clear schism in the percentage fluid overload between survivors and non-survivors in the current report originating from the same institution as the 2001 paper might reflect a drift in practice where CRRT is started earlier, thus avoiding a higher initial degree of fluid overload. Our patients had an average of 15% fluid overload at CRRT start, with a shift between eras from 17 to 14%, although not statistically significant. The time to CRRT initiation seemed shorter in the modern era, although this difference was not statistically significant. The difference in percentage fluid overload might at least in part reflect a more careful attention to fluid balance by the general healthcare team.

Our results confirm that patients requiring treatment with CRRT are rarely patients with primary renal disease; rather they are patients who secondarily suffer from kidney injury in the context of their primary disease condition and comorbidities. In fact, half of the patients treated across both eras were immunocompromised, including cancer, organ transplantation, and autoimmune conditions. Patients who have undergone stem cell transplants and received renal replacement therapy have been studied, but little has been studied to date surrounding the immune status of patients requiring CRRT [5, 40, 41]. While the difference in survival did not achieve statistical significance, we observed a trend towards decreased survival rates amongst patients who were immunocompromised. There is very limited pharmacokinetic data in pediatric CRRT, potentially impacting optimal treatment of CRRT patients particularly with antimicrobials, as pediatric CRRT patients are reported to have increased infections [42]. Further study is warranted to determine if higher infection susceptibility translates to increased morbidity from suboptimal treatment.

Our patient population showed no difference in survival based on the total time spent on CRRT, examined both as total median days on CRRT and when categorized incrementally. This is consistent with reporting from the ppCRRT registry, as well as a much smaller study of 39 patients, for whom there was no significant difference in survival rates based on duration of CRRT for greater than or less than 4 weeks [6, 43]. Across all groupings in our study, survivors consistently demonstrated shorter times from both hospital and ICU admissions to initiation of CRRT compared to non-survivors, but an overall shorter hospital length of stay. In pediatrics, Hayes et al. showed no difference in the number of hospital days prior to starting CRRT, however Modem et al. did find a significantly longer time to CRRT initiation amongst non-survivors [14, 21]. Adult CRRT investigations have evaluated the benefits of early versus late start CRRT, defined by timing to initiate CRRT in the setting of AKI severity, without any clearly demonstrable benefit [44–46]. We suspect differential survival outcomes with respect to time to initiation of CRRT may be more indicative of severe, progressive multi-organ illness with eventual AKI development rather than a beneficial impact of earlier CRRT initiation on outcomes. While percentage of fluid overload at initiation of CRRT has been linked to longer ventilation times [21], ventilation time alone in pediatric patients treated with CRRT has not been previously evaluated for survival, and suggests increased severity of illness associated with patient mortality.

For those patients who survive their hospital course that includes CRRT, we found that overall only 44% of patients were subsequently seen for dedicated outpatient nephrology care, however between eras, that number improved from 33 to 54%. Only one-third of pediatric and adult patients follow up with a nephrologist after an episode of AKI [47, 48]. While the exact time course for developing chronic kidney disease (CKD) is unknown, a progressively higher rate of CKD is observed over time amongst AKI survivors [47, 49]. In pediatric patients with CKD, increasing proteinuria is associated with decreased renal function, and excellent blood pressure control has been correlated with slowed progression of renal disease [50, 51]. Thus, early intervention is critical in slowing the relentless progression of CKD, and patients whose course of critical illness has included AKI may benefit from dedicated longitudinal follow-up with a nephrologist for monitoring of blood pressure, urinary protein, and kidney function. More importantly, this finding underscores the need to educate fellow physicians, patients, and parents on the importance of monitoring kidney function and maintaining nephrology follow-up over the patient’s lifetime.

Our study has several limitations. The retrospective nature of the study did not allow us to determine certain important patient characteristics, such as severity of illness indices, which would have been helpful in refining the definition of the cohorts and to examine if indeed sicker patients were being treated with CRRT in the latter era, perhaps accounting for the lack of improvement in survival, at least partially. We were also only able to determine outpatient follow-up in patients who came to our nephrology clinic, and may have missed some patients who were followed at other centers. Lack of an AKI/CRRT survivors’ clinic or a formal outreach program where these patients are being followed in conjunction with primary care physician is partially responsible for the incomplete follow-up.

The largest and most comprehensive evaluation of pediatric AKI was recently published, demonstrating an AKI rate of 26.9% in pediatric ICU around the world [52]. Studies have shown that AKI, regardless of the criterion scoring used and with or without provision of CRRT, portend the greatest risk for mortality [18, 53, 54]. Since the ppCRRT registry was concluded more than ten years ago, the pediatric nephrology and critical care communities gained a wealth of information, including data on the demographics, epidemiologic, and technical aspects of pediatric CRRT [25]. The field of AKI clinical research has exploded in the last decade, improving our understanding of the impact of AKI on survival as well as other morbidity such as new disability [55]. While feasibility of pediatric CRRT was still being questioned in the early 2000s, it has now become the standard of care. Our study provides evidence for a need to continue performing interval assessments of this patient population, especially with advances across critical care medicine, not limited to renal replacement devices. It is especially disheartening to see that mortality rate has not decreased at all despite technological advances and accumulating clinical experience. The reasons underlying this observation require further exploration. While it is certainly possible that we are treating sicker patients with CRRT, in the absence of severity of illness indicators, we cannot conclude this to be the only reason. Lack of specialized renal replacement equipment for pediatric patients, uncertainty about optimal antibiotic dosing on CRRT, and under nutrition likely all play roles in little improvement seen in survival among patients treated with CRRT during their hospital course. While we benefit from good patient volume numbers, this is still a single-center study, reflecting the unique aspects of CRRT prescription and delivery at one location. Multi-center assessments to amass collaborative patient evaluations will continue to help further assess multi-layered aspects of patient care, including a clarification of who is most affected by fluid overload, changing demographics of the population and their needs, and how duration of CRRT correlates with patient outcomes. Additionally, longitudinal care of these patients who survive with AKI is for maintaining maximal health in the face of progressive chronic kidney disease.

## Conclusions

Over the last decade, the demographics of our pediatric CRRT population have shifted slightly to younger and smaller patients, but overall diagnostic categories and outcomes have not changed. Although nephrology outpatient follow-up in AKI survivors has improved over time, it remains poor at around 50%. Further studies focusing on pediatric CRRT patients to examine areas of limited data such as pharmacokinetics, dose delivery, quality indicators, and renal recovery will enhance our practice of CRRT and might improve outcomes.
